# Paxillin-Y118 phosphorylation contributes to the control of Src-induced anchorage-independent growth by FAK and adhesion

**DOI:** 10.1186/1471-2407-9-12

**Published:** 2009-01-12

**Authors:** Sanjay Sachdev, Yahao Bu, Irwin H Gelman

**Affiliations:** 1 Department of Cancer Genetics, Roswell Park Cancer Institute, Elm and Carlton Streets, Buffalo, NY 14263, USA

## Abstract

**Background:**

Focal adhesion kinase (FAK) and Src are protein tyrosine kinases that physically and functionally interact to facilitate cancer progression by regulating oncogenic processes such as cell motility, survival, proliferation, invasiveness, and angiogenesis.

**Method:**

To understand how FAK affects oncogenesis through the phosphorylation of cellular substrates of Src, we analyzed the phosphorylation profile of a panel of Src substrates in parental and v-Src-expressing FAK+/+ and FAK-/- mouse embryo fibroblasts, under conditions of anchorage-dependent (adherent) and -independent (suspension) growth.

**Results:**

Total Src-induced cellular tyrosine phosphorylation as well as the number of phosphotyrosyl substrates was higher in suspension versus adherent cultures. Although the total level of Src-induced cellular phosphorylation was similar in FAK+/+ and FAK-/- backgrounds, the phosphorylation of some substrates was influenced by FAK depending on adherence state. Specifically, in the absence of FAK, Src induced higher phosphorylation of p190RhoGAP, paxillin (poY118) and Crk irrespective of adhesion state, PKC-δ (poY311), connexin-43 (poY265) and Sam68 only under adherent conditions, and p56Dok-2 (poY351) and p120catenin (poY228) only under suspension conditions. In contrast, FAK enhanced the Src-induced phosphorylation of vinculin (poY100 and poY1065) and p130CAS (poY410) irrespective of adherence state, p56Dok-2 (poY351) and p120catenin (poY228) only under adherent conditions, and connexin-43 (poY265), cortactin (poY421) and paxillin (poY31) only under suspension conditions. The Src-induced phosphorylation of Eps8, PLC-γ1 and Shc (poY239/poY240) were not affected by either FAK or adherence status. The enhanced anchorage-independent growth of FAK-/-[v-Src] cells was selectively decreased by expression of paxillin^Y118F^, but not by WT-paxillin, p120catenin^Y228F ^or Shc^Y239/240F^, identifying for the first time a role for paxillin^poY118 ^in Src-induced anchorage-independent growth. Knockdown of FAK by siRNA in the human colon cancer lines HT-25 and RKO, resulted in increased paxillin^poY118 ^levels under suspension conditions as well as increased anchorage-independent growth, supporting the notion that FAK attenuates anchorage-independent growth by suppressing adhesion-dependent phosphorylation of paxillin^Y118^.

**Conclusion:**

These data suggest that phosphorylation of Src substrates is a dynamic process, influenced temporally and spatially by factors such as FAK and adhesion.

## Background

Autophosphorylation of the focal adhesion kinase, FAK, at Y397 upon integrin-mediated activation produces an SH2-mediated binding site for Src-family tyrosine kinases, or alternatively, other signaling proteins such as phosphatidylinositol 3-kinase (PI3K), Shc, phospholipase-Cγ or Grb7 [1,2]. Once bound, Src can phosphorylate FAK on several residues including Y925 resulting in the further activation of FAK tyrosine kinase activity [3] and in the phosphorylation of many other cellular substrates. FAK also encodes ligands for multiple protein docking domains such as SH3, an N-terminal FERM domain that facilitates association with integrins and growth factor receptors, and a C-terminal domain that facilitates paxillin/talin binding and focal adhesion targeting (reviewed in ref. [1,4,5]). Indeed, the maximal tyrosine phosphorylation of many cellular substrates, such as paxillin, requires both Src and FAK activity [6]. Current thinking suggests that the mutual activation of Src and FAK in response to growth factors, chemotactic agents and cell adhesion leads to activation of a number of downstream pathways in a spatially and temporally controlled manner [7].

Both FAK and Src have been implicated as playing major roles in cancer progression, especially relating to metastatic potential. For example, activation of Src-family kinases has been reported in many primary cancers such as those affecting the colon, GI tract, breast and brain (reviewed in ref. [8]), and activated Src seems to play a critical role in the recruitment of endothelial cells to sites of tumor angiogenesis [9-11]. FAK protein and activation levels are increased in many primary cancers and further increased in metastatic lesions [12-23]. Moreover, the loss of FAK activity or expression suppresses metastatic progression in tumor xenograft models, underlining an important positive role for FAK in the development of malignancy [24].

Paradoxically, there are a growing number of studies correlating lower FAK levels with poorer patient survival [25,26], suggesting that FAK may actually attenuate some of the lethal parameters of cancer progression.

As FAK and Src interact physically and functionally, it was expected that FAK-null cells would be defective for oncogenic transformation by v-Src. Thus, it was surprising that in the absence of FAK, v-Src could induce morphological transformation, cell motility, cell polarity and Matrigel invasiveness [27,28], and even enhance the frequency of anchorage-independent growth 5- to 10-fold [27]. Indeed, a high-resolution one-dimensional anti-phosphotyrosine analysis of FAK+/+ versus FAK-/- fibroblasts expressing v-Src identified fewer than 10 varying substrates. Although the full phosphorylation of some cellular substrates, such as Endophilin A2, requires both FAK and Src [29], it is possible that such substrates are dispensable for oncogenic transformation. A further complication is that phosphorylation of some substrates, such as PI3K, varied only under conditions of anchorage independence [30]. Indeed, we demonstrated that the superactivation of PI3K was required and sufficient for the enhanced v-Src-induced anchorage-independent growth of FAK-/- fibroblasts [30].

Here, we analyze how FAK affects the phosphorylation of a panel of known Src substrates under conditions of anchorage-dependent and -independent growth. Whereas the majority of substrate phosphorylations neither required FAK for v-Src-induced phosphorylation nor were enhanced in the presence of FAK, we identified a small number of substrates whose phosphorylation was affected by FAK and/or adherence state. These variations suggest that FAK and integrins might play either positive or negative roles during the overall process of malignant progression.

## Methods

### Cell lines and growth conditions

FAK+/+ and FAK-/- mouse embryo fibroblasts (MEF) from a p53-/- lineage [31] transduced with v-Src or empty vector containing puro^r ^gene [30] were maintained in Dulbecco's modified Eagle's medium (DMEM) supplemented with 10% heat-inactivated bovine serum (BS). For suspension growth, cells were plated onto bacterial culture plates pre-coated with 1% agarose and then incubated in DMEM/10% BS for 1 d before harvesting. Trypan blue exclusion staining indicated <5% decrease in cell viability under these conditions compared to anchorage-dependent growth (data not shown). FAK knockdown in human colon cancer cell lines was performed by transfecting double-stranded 10 nM FAK siRNA [32] or GFP siRNA (5'- GGAGCGCACCAUCUUCUUCUU-3') [33] with Lipofectamine for 6 hours in media containing BS, followed by two washes with PBS and continued incubation in complete media. After two days, aliquots of the cells were plated in soft agar for AIG assays, and after one more day of incubation in culture, aliquots of the cells were analyzed by IB FAK, paxillin, paxillin^poY118 ^and GAPDH levels.

### Protein isolation

FAK+/+, FAK-/-, FAK+/+[v-Src] and FAK-/-[v-Src] cells grown under adherent or suspension conditions were rinsed with cold PBS and lysed in cold RIPA buffer (10 mM Tris-HCl, pH 7.4, 0.15 M NaCl, 5 mM EDTA, 8% glycerol, 1% Triton X-100, 0.1% SDS and 0.5% sodium deoxycholate) supplemented with Complete Mini protease inhibitor cocktail tablets (1 tablet/10 ml RIPA; Roche Diagnostics, Alameda, CA) and phosphatase inhibitors (1 mM sodium orthovanadate and 10 mM sodium fluoride) for 30 min. After centrifuging at 16,000 × g for 15 min at 4°C to remove debris, protein concentrations were determined using Bradford dye-binding assay (Bio-Rad, Hercules, CA).

### Immunoblotting (IB) analysis

RIPA lysates containing 60 μg of cell protein were boiled in laemmli buffer for 3 min, separated using 4–15% gradient SDS-polyacrylamide gels (BioRad), electrophoretically transferred on to PVDF membrane (Perkin-Elmer, Wellesley, MA) in transfer buffer (25 mM Tris-base, 192 mM glycine and 20% methanol) and immunoblotted (IB) with various antibodies (Ab), as previously described [34]. Membranes were blocked for 1 h at room temperature with either 3% bovine serum albumin or 5% non-fat dry milk; the choice of blocking agent was determined empirically so as to minimize the appearance of non-specific protein bands while not diminishing the signal of the Src substrate. The primary phospho-Abs used, described in Table 1A, were purchased from Invitrogen/BioSource (Carlsbad, CA), Cell Signaling Technology, Inc. (Danvers, MA), Santa Cruz Biotechnology (Santa Cruz, CA) or BD Biotechnology (San Jose, CA), with the exception of Ab to FAK (Chemicon, Temecula, CA). The secondary Abs used were horseradish peroxidase conjugated anti-rabbit, anti-mouse or anti-goat IgG (Chemicon). After treating with Lumi-Light chemiluminescence substrate (Roche), Ab binding was imaged and quantified using GeneTools software on a Chemi-Genius^2 ^bioimaging system (Syngene, Frederick, MD). If required, membranes were stripped and reprobed as described previously [35]. Note that v-Src typically decreases actin 2-fold and increases GADPH 2-fold [27,36] and thus, protein-loading normalization incorporated these variations.

**Table 1:** Antibodies used in this study

(A) Phospho-specific antibodies				
Substrate	poY location	Mol mass (kDa)	Co.; Cat. #^a^	MM/RP/GP^b^
cortactin	421	80/85	BioS; 44–854	RP
paxillin	31	68	BioS; 44–720	RP
paxillin	118	68	BioS; 44–722	RP
Shc	239/240	46, 52, 66	BioS; 44–830	RP
PKC-δ	311	77	BioS; 44–950	RP
PLC-γ1	783	130	Bios; 44–696	RP
p56Dok-2	351	56, 58	CS; 3911	RP
CAS	410	130	CS; 4011	RP
connexin-43	265	43	SC; sc-17220	GP
p120catenin	228	120	BD; 612536	MM
vinculin	100	130	BioS; 44–1074G	RP
vinculin	1065	130	BioS; 44–1078G	RP
(B) Antibodies used for IP				
**Substrate**	**Mol mass (kDa)**	**Co.; Cat. #**^a^	**MM/RP**^b^	
	
Sam68	68	SC; sc-333	RP	
	
Annexin II (H-50)	36(90)	SC; sc-9061	RP	
	
p190Rho-GAP	190	BD; 610149	MM	
	
Eps8	97	BD; 610143	MM	
	
Crk (Crk II)	40	BD; 610035	MM	

(C) Antibodies used for total substrate protein level				
**Substrate**	**Mol mass (kDa)**	**Co.; Cat. #**^a^	**MM/RP**^b^	
	
cortactin	80/85	CS; 3502	RP	
	
paxillin	68	BD; 610551	MM	
	
Shc	46, 52, 66	CS; 2432	RP	
	
PKC-δ	77	SC; sc-213	RP	
	
PLC-γ1	130	CS; 2822	RP	
	
p56Dok-2	56, 58	CS; 3914	RP	
	
CAS	130	BD; 610271	MM	
	
p120catenin	120	BD; 610133	MM	
	
vinculin	130	Sigma; V-9131	MM	
	
GAPDH	37	SC; sc-25778	RP	

(D) Epitope-tag antibodies				
**Epitope tag**	**Co.; Cat. #**^a^	**MM/RP**^b^		
		
GST	SC; sc-138	MM		
		
FLAG	Sigma; F3040	MM		
		
GFP	Invitrogen; A6455	RP		

^a ^BioS – BioSource; CS – Cell Signaling; SC – Santa Cruz; BD – BD Biosciences

^b ^Abs: RP – Rabbit polyclonal; MM – Mouse monoclonal; GP – Goat polyclonal

* Abs to actinin (Sigma; A5044), CAS^poY165 ^(CS; 4015), CAS^poY249 ^(CS; 4014), p120catenin^poY96 ^(BD; 612534), p120catenin^poY280 ^(BD; 612538), ezrin^poY353 ^(CS; 3144) did not recognize appropriately-sized bands.

### Immunoprecipitation (IP) analysis

IP with various Abs (Table 1B) was carried out essentially as described previously [30] using 1 μg of Ab per 100 μg of protein lysates plus pre-washed Protein A/G PLUS agarose (Santa Cruz). The IB step used mAb-4G10 (Upstate/Millipore, Billerica, MA) at 1:5000 using secondary Ab and signal quantification described above.

### Stable transfection

Puromycin-resistant FAK+/+[v-Src] and FAK-/-[v-Src] cells were transfected using Lipofectamine 2000 (Invitrogen) with the expression plasmids, pEBG-ShcY239/240F (GST tag; gift of Kodi Ravichandran, University of Virginia), pFLAG2AB-cateninY228F (below) or pEGFP-paxillinY118F (gift of Alan Horwitz, University of Virginia) together with the vector pTRE2-hygro (gift of Andrei Bakin, Roswell Park Cancer Institute) used as a hygromycin-selection marker. Colonies were selected in DMEM containing 500 μg/ml hygromycin (Roche), expanded, and then the expression of the exogenous proteins was verified by Western blotting using Abs specific for the epitope tags (GST, FLAG or GFP). The pFLAG2AB-cateninY228F expression plasmid was generated by amplifying the mouse catenin1A^Y228F ^coding sequence from pRc/RSV-mctn-1A/228F (gift of Albert Reynolds, Vanderbilt University) and then subcloning into pcDNA3-FLAG2AB vector (gift of Scott Weed, West Virginia University) cut with Kpn1 and EcoRI.

### Anchorage-independent growth

Growth in soft agar was assayed in 60-mm dishes prepared with a lower layer of 0.7% agar in DMEM/10% BS overlaid with top agarose (0.4%)/DMEM/10% BS containing 10^4 ^suspended cells. Cells were fed every 3 days with fresh culture medium. 3 weeks after plating, colonies were stained with 3-(4,5-dimethylthiazol-2-yl)-2,5-diphenyl tetrazolium bromide (Sigma) and counted.

### Clonogenic assay

Cell viability was analyzed by seeding 4 × 10^2 ^cells into 100-mm dishes and then counting colonies after 10 days of culture. Colonies were fixed and stained using Diff-Quik Stain Set (DADE EBHRING, Newark, DE) according to manufacturer's instructions, and then counted in triplicate.

## Results and Discussion

**Figure 1:**
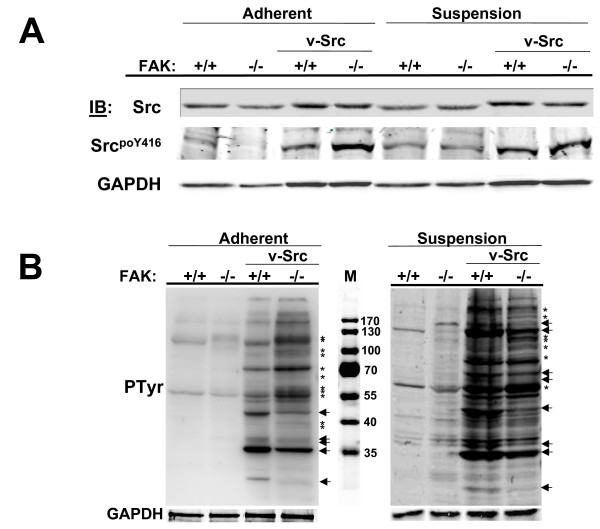
**FAK- and adhesion-effects on v-Src substrate choice**. (**A**) FAK+/+[puro], FAK-/-[puro], FAK+/+[v-Src] and FAK-/-[v-Src] cells grown in adherent or suspension conditions as described in Experimental Procedures were analyzed by IB for levels of total Src, Src^poY416 ^autophosphorylation or GAPDH (as a loading control). [Note that the decrease in Src protein, Src^poY416 ^and GAPDH levels in lane 2 (second from left) is not reproducible; relative Src activation levels in adherent FAK-/-[v-Src] cells are typically comparable to those in adherent FAK+/+[v-Src] cells]. (**B**) Anti-phosphotyrosine (MAb4G10) IB from equal protein loads of FAK+/+[puro], FAK-/-[puro], FAK+/+[v-Src] and FAK-/-[v-Src] cell lysates. M, proteins markers in kDa. Decreased Src-induced tyrosine phosphorylation events in the absence of FAK are marked by arrows whereas increased tyrosine phosphorylation events in the absence of FAK are marked by asterisks. These data are typical of at least three independent experiments. A GAPDH IB is shown below as a loading control.

Many studies have demonstrated physical and functional interactions between FAK and Src in response to integrin- and growth factor-mediated signals. However, most of the studies linking FAK/Src complexes with cell motility, proliferation and cell survival in cancer progression involve adherent cell populations. Yet, many parameters of cancer biology *in vivo*, especially metastasis, require anchorage-independent proliferation. To elucidate the roles of adhesion and/or FAK on Src-induced oncogenesis, we developed FAK+/+ or FAK-/- MEF expressing relatively similar protein and activity levels of the v-Src oncogene (Fig. 1A). As we reported previously [27], the relative level of v-Src autophosphorylation (poY416) under adherent conditions was consistently 2-fold higher in the FAK-/- background, and this correlated with a slightly higher overall level of cellular phosphotyrosyl proteins compared to the FAK+/+ background (Fig. 1B, left panel). Interestingly, overall v-Src-induced tyrosine phosphorylation of cellular substrates, as well as the total number of substrates, was slightly higher in v-Src cells kept in suspension, irrespective of FAK content (Fig. 1B, right panel). This was not due to relative increases in Src activation levels in the suspended cells (Fig. 1A). Although the gross level of Src-induced tyrosine phosphorylation is similar in the presence or absence of FAK, there are a small number of substrates whose relative phosphorylation level is either increased or decreased by FAK (Fig. 1B). Moreover, in adherent cells, there were more phosphorylated Src substrates in the absence of FAK (stars), whereas in the suspended cells, there were a roughly equal number of substrates favored in the presence (arrows) or absence (stars) of FAK. These data are consistent with the notion that FAK modulates the ability of activated Src to associate with and/or phosphorylate specific cellular substrates [27].

The possibility that variations in substrate choice by v-Src could be influenced by FAK and/or adhesion may have consequences on specific parameters of oncogenic transformation. For example, v-Src induces 5- to 10-fold higher anchorage-independent growth (AIG) in the absence of FAK, correlating with a concomitant increase in PI3K activation levels under AIG conditions [27,30]. However, we showed previously that the twofold higher level of activated v-Src is not responsible for the enhanced AIG (eAIG) in the FAK-/- background inasmuch as varying v-Src levels only altered soft agar colony size [30]. In order to clarify roles for FAK and adhesion in regulating v-Src-induced phosphorylation of cellular substrates, FAK+/+, FAK-/- MEFs and their v-Src transformed derivatives, FAK+/+[v-Src] and FAK-/-[v-Src], were incubated in the presence of serum under conditions of adherence versus suspension, and then cell lysates were probed by IB for changes in the phosphorylation status of known Src substrates. Thus, IBs were probed with either phospho-specific substrate Abs (Table 1A), or alternatively, substrate proteins were immunoprecipitated and then analyzed by anti-PTyr IB using MAb-4G10 (Table 1B). In all cases, the change in the relative phosphorylation state of each substrate was normalized to apo-protein levels as well as to GAPDH, used as a loading control. One caveat is that GAPDH levels typically increase twofold in murine cells transformed with v-Src [30,36], a finding consistent throughout the current study, and this was factored into the final normalization.

**Figure 2:**
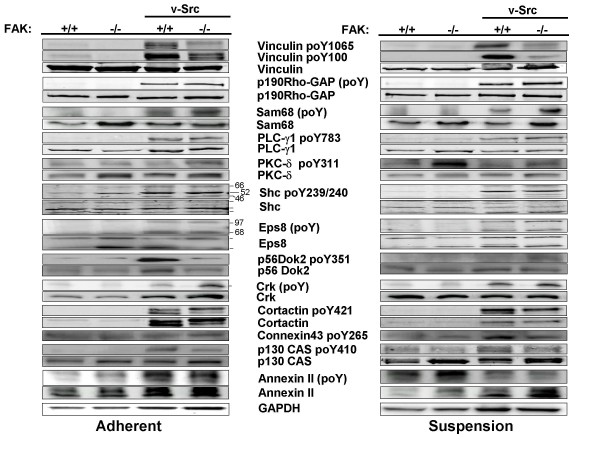
**FAK and adhesion modulate v-Src-induced phosphorylation of various Src substrates**. **(A) **Lysates from adherent cultures of FAK+/+[puro], FAK-/-[puro], FAK+/+[v-Src] and FAK-/-[v-Src] cells were probed either directly by IB for specific phosphorylated form(s) of the Src substrate proteins, total substrate protein levels or GAPDH, or probed for total phosphorylated protein by immunoprecipitating with substrate-specific Ab followed by IB for phosphotyrosine using MAb4G10. **(B) **Same IB or IP/IB analysis as in panel A using lysates of suspension cultures. Each of these blots is typical of at least duplicate independent experiments.

Table 2 shows that only a minority of the Src substrates studied were unaffected by FAK and/or adhesion. In fact, only the phosphorylation signals of Eps8, PLC-γ1 (at Y783), and Shc (at Y239/240) showed no change under these variables (Fig. 2). The phosphorylation of cortactin (at Y421) and paxillin (at Y31) was unaffected by FAK only in adherent cultures, whereas the phosphorylation of Sam68 and PKC-δ (at Y311) was unaffected by FAK only in suspension cultures. Interestingly, we observed a v-Src-induced increase in cortactin protein levels, and moreover, a slower-migrating form whose abundance was increased even more in FAK-/-[v-Src] cells. The phosphorylation of several Src substrates was enhanced by FAK, irrespective of adherence state. These include vinculin (at both the Y100 and Y1065 sites) and CAS. In contrast, the phosphorylation of p190RhoGAP, paxillin (at Y118) and Crk was favored in the absence of FAK, irrespective of adherence state. Lastly, although v-Src induced annexin II protein levels, the phosphorylation of annexin II seemed to be inhibited by v-Src in the absence of FAK.

**Table 2:** Relative Phosphorylation Level of Src Substrates in FAK+/+[Src] vs. FAK-/-[Src] Cells

	Adherent			Suspension		
**v-Src Substrates**	Similar level^*a*^	More in FAK+/+ [v-Src]	More in FAK-/- [v-Src]	Similar level	More in FAK+/+ [v-Src]	More in FAK-/- [v-Src]

Vinculin (poY100)		5 – 6			7 – 8	

p190RhoGAP (poY)*			1.5 – 2			1.5 – 2

Shc (poY239/poY240)	Yes			Yes		

Sam68 (poY)*			2	Yes		

PLC-γ1 (poY783)	Yes			Yes		

PKC-δ (poY311)			2	Yes		

Paxillin (poY118)			4			10

Paxillin (poY31)	Yes				2 – 3	

Eps8 (poY)*, ^*b*^	Yes			Yes		

p56 Dok-2 (poY351)		5				2

Crk (poY)*			2			2

Cortactin (poY421)^*c*^	Yes				2	

Connexin43 (poY265)^*d*^			2		2–3	

p120Catenin (poY228)		2				2

p130 CAS (poY410)		2			2	

Annexin II (poY)*	Yes			Yes		[3]^*e*^

*blotting with mAb 4G10 anti-PTyr was used to ascertain the level of tyrosine phosphorylation after immunoprecipitation with substrate-specific Abs.

^*a *^statistically equal level of relative substrate phosphorylation between FAK+/+[Src] and FAK-/-[Src] cells, based on triplicate experiments. Relative phosphorylation for a given experiment is calculated as the phospho- substrate signal normalized to total substrate protein level, then normalized to loading control proteins. Note that Src transformation increases GAPDH protein levels ~2-fold and decreases actin protein level ~2-fold in the FAK+/+ background in adherent cells only.

^*b *^for both 97 kDa and 68 kDa Eps8 isoforms.

^*c *^in adherent cultures, the 80 kDa cortactin isoform protein level is reduced in the absence of FAK, but the relative phosphorylation levels are similar.

^*d *^connexin-43 (poY265) levels were not normalized.

^*e *^relative *decrease *in phospho-annexin II levels in suspended FAK-/-[v-Src] cells.

**Figure 3:**
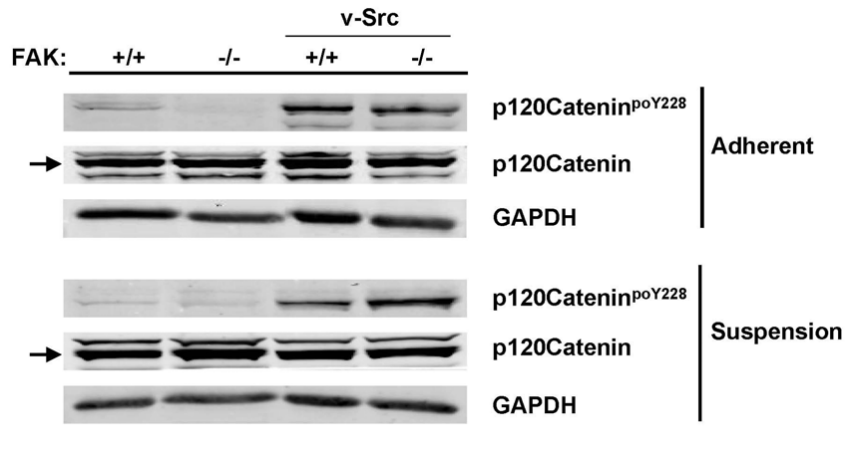
**FAK and adhesion modulate v-Src-induced phosphorylation of p120catenin**. Lysates from adherent or suspension cultures of FAK+/+[puro], FAK-/-[puro], FAK+/+[v-Src] and FAK-/-[v-Src] cells were probed by IB for total p120catenin, p120catenin^poY228 ^or GAPDH. These data are typical of at least three independent experiments. *Arrows*, p120catenin identified by the p120catenin^poY228^-specific Ab.

**Figure 4:**
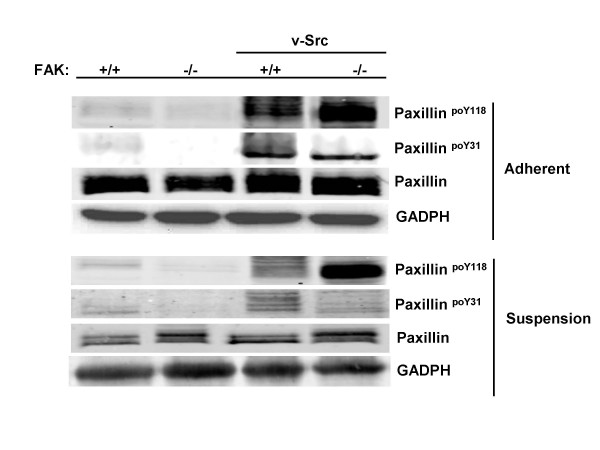
**Enhanced v-Src-induced paxillin phosphorylation is attenuated by FAK**. Lysates from adherent or suspension cultures of FAK+/+[puro], FAK-/-[puro], FAK+/+[v-Src] and FAK-/-[v-Src] cells were probed by IB for total paxillin, paxillin^poY31^, paxillin^poY118 ^or GAPDH. These data are typical of at least three independent experiments.

The phosphorylation of a set of Src substrates was influenced by FAK depending on adhesion conditions. Specifically, the connexin43^poY265 ^signal was enhanced by FAK in suspension cultures, yet attenuated by FAK in adherent cultures (Table 2; Fig. 2). Conversely, the p120 catenin^poY228 ^(Fig. 3) and p56Dok-2^poY351 ^signals (Fig. 2) were enhanced by FAK in adherent cultures, yet attenuated by FAK in suspension cultures. The Src-induced phosphorylation of Sam68 was enhanced in the absence of FAK only in adherent cultures (Fig. 2). Lastly, the Src-induced phosphorylation of paxillin at Y118 in the absence of FAK is enhanced to an even greater extent in suspension cultures (10- versus 4-fold, respectively; Fig. 4). Taken together, our data indicate that the phosphorylation of most of the well-documented Src substrates is influenced by FAK and/or adhesion.

**Figure 5:**
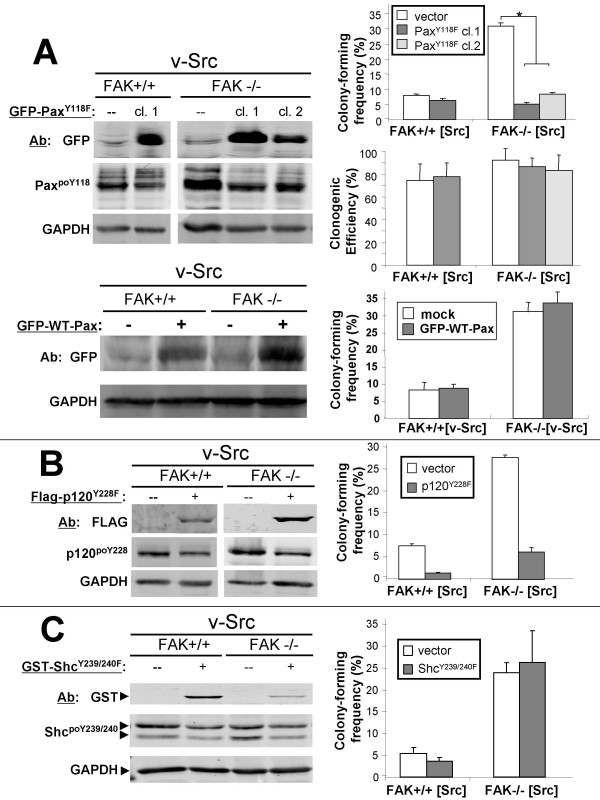
**Phosphorylation of paxillin^poY118 ^is required for enhanced AIG by FAK-/-[v-Src] cells**. (**A**) Left panel- IB analysis of FAK+/+[v-Src] or FAK-/-[v-Src] cell clones ("cl.") stably transfected with empty vector (--) or a GFP-paxillin^Y118F^-expressing vector, probed with Abs specific for GFP, paxillin^poY118 ^or GAPDH. Aliquots of these cells were analyzed by anchorage-independent growth (top right) or for clonogenic efficiency (bottom right) as described in Materials and Methods. Error bars, S.E. *, P < 0.01. (**B**) A similar analysis as in panel A except on cells stably expressing FLAG-p120^Y228F^, with IBs probed for FLAG, p120catenin^poY228 ^or GAPDH. Note that there is no statistical difference in the p120catenin^Y228F^-mediated decrease in AIG between the FAK+/+[v-Src] and FAK-/-[v-Src] cells. (**C**) A similar analysis as in panel A except on cells stably expressing GST-Shc^Y239/240F^, with IBs probed for GST-tag, Shc^poY239/240^, or GAPDH.

It is possible that the eAIG induced by v-Src in the absence of FAK is mediated by the enhanced phosphorylation of p120catenin or paxillin, Src substrates whose phosphorylation was consistently increased in FAK-/-[v-Src] cells. To address this, stable FAK+/+[v-Src] and FAK-/-[v-Src] cell clones were produced that express p120catenin^Y228F ^or paxillin^Y118F ^alleles. As a negative control, the cells were transfected with Shc^Y239/240F^, whose phosphorylation is affected by neither FAK nor adherence status (Table 2). Figs. 5A–C show the expression of these exogenous proteins (probed for their epitope tags). As expected, the expression of these non-phosphorylatable mutants suppressed the levels of endogenous Shc^poY239/240^, p120catenin^poY228 ^or paxillin^poY118^. This effect was apparent in multiple cell clones, and, in FAK-/-[v-Src] cells transiently co-transfected with paxillin^poY118 ^and pEGFP (sorted for GFP-positive cells; data not shown), strongly suggesting that the Y->F mutants function as dominant-interfering alleles. We cannot rule out, however, that these mutants also affected the phosphorylation of other residues on their endogenous counterparts. Importantly, the ectopic expression of paxillin^Y118F^, but not p120catenin^Y228F ^or Shc^Y239/240F^, selectively decreased the *enhanced *AIG in FAK-null cells compared to those expressing FAK (Fig. 5, panels A-C). This was not due to changes in cell survival based on the similar clonogenic frequencies between FAK+/+ or FAK-/- v-Src clones expressing the paxillin^Y118F ^allele (Fig. 5A, right middle panel). As a further control, we showed that the stable expression of the WT-paxillin allele (expressed as a GFP-paxillin fusion) failed to alter eAIG in FAK-/-[v-Src] cells (Fig. 5A, bottom right). This strongly suggests that the enhanced Src-induced phosphorylation of paxillin at Y118 in the absence of FAK plays a significant role in inducing enhanced Src-mediated AIG. This contrasts with the finding that Src-induced phosphorylation of p120catenin on Y228, but not of Shc on Y239/240, was critical to Src-induced anchorage-independent growth irrespective of the FAK background (Fig. 5B &5C).

**Figure 6:**
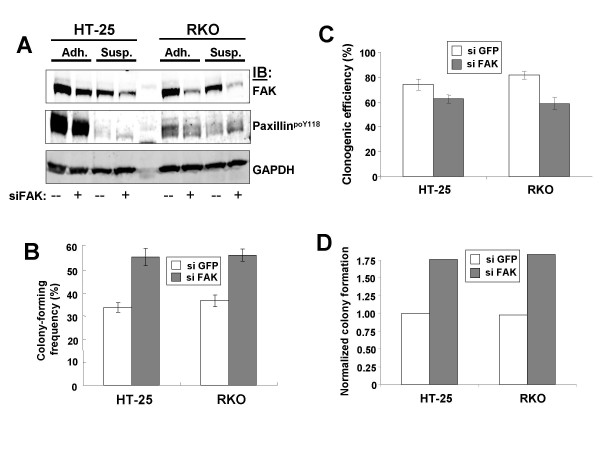
**Loss of FAK in human colon cancer cell lines leads to increased AIG and paxillin^poY188 ^accumulation under suspension growth conditions**. (**A**) IB analysis of HT-25 and RKO colon cancer cells grown under adherent or suspension conditions that were incubated with either FAK or GFP siRNA for 72 h, probed for FAK, paxillin (not shown) paxillin^poY118 ^or GAPDH. Note that paxillin protein levels did not change under these conditions. Aliquots of the cells in panel A were analyzed for anchorage-independent growth (panel **B**) or for clonogenic efficiency (panel **C**) as described in Materials and Methods. Error bars, S.E. from triplicate plates in two independent experiments. *p *< 0.01. (**D**) Normalized AIG, based on the mean of the AIG data from panel B normalized to the mean of the survival data in panel C.

Given the artificial nature of our cell system, we sought to determine whether the relationship between loss of FAK, increased paxillin^poY118 ^and eAIG was manifest in human cancer cells lines. Colon cancer is marked by the activation of Src, mainly through overexpression although small percentages encode mutated Src known to cause upregulation of its intrinsic tyrosine kinase activity [37]. Because the knockdown of FAK in cancer cell lines using antisense oligonucleotides or siRNAs often induces apoptosis [27,38-42], we screened a panel of human colon cancer cell lines for those that failed to apoptose after treatment with FAK siRNA. Treatment of two such lines, HT-25 and RKO, with FAK-siRNA under conditions of adherent or suspension growth caused 3- to 4-fold decreases in FAK protein level (Fig. 6A). The loss of FAK marginally decreased cell survival as measured by clonogenic colony growth (Fig. 6C), yet resulted in significant increases in AIG as measured by soft agar colony-forming frequencies (Fig. 6B). Note that the loss of FAK did not affect the colony size formed by these cell lines (not shown). If the AIG frequency is normalized to cell survival rates, FAK knockdown induces an equivalent 1.75-fold increase in relative AIG in both HT-25 and RKO over control cells (GFP siRNA). Given that the loss of FAK in these cancer cell lines recapitulated the eAIG found in our FAK-/-]v-Src] MEF, we analyzed how the loss of FAK affected paxillin^poY118 ^levels under adherent or suspension conditions. Suspension cells treated with siFAK exhibit increased paxillin^poY118 ^levels, especially a fast-migrating isoform, whereas the loss of FAK in adherent cells resulted in decreased relative paxillin^poY118 ^levels (Fig. 6A). The protein levels of GAPDH (Fig. 6A) or paxillin (not shown) did not alter by these conditions. These data strengthen the notion that in HT-25 and RKO, FAK may antagonize AIG by inhibiting the phosphorylation of paxillin^Y118 ^by activated Src-family kinases.

Cancer progression, especially metastasis, is controlled by multiple stages, with varying dependencies on the proliferative, motility and survival signals mediated by cell adhesion and FAK [1,43]. For example, primary and metastatic site tumors cells require growth conditions less dependent on integrin-mediated adhesion than individual metastatic cells require while intravasating from primary lesion and extravasating to peripheral sites [44]. Similarly, the motility-promoting activity of FAK would be more desirable to facilitate extravasation and intravasation of metastatic cells [45], yet once at peripheral sites, continued FAK motility signals would likely be suppressed in the growing metastatic lesion. Although the activation of Src-family kinases has been noted as an important event in early cancer progression- mostly involved in mediating proliferative signals- there is mounting data that Src signaling is especially critical for the metastatic process, specifically, to facilitate cancer cell survival and neovascularization of metastatic sites [8,9,11]. Our results indicate that in a majority of well-documented Src substrates, Src-induced phosphorylation is influenced by FAK and/or adhesion. Some are FAK dependent whereas as others are attenuated by FAK, irrespective of adherence state. Yet others are affected by FAK only under adherent or non-adherent conditions. Because our cells were derived in a p53-/- background [31], it would be interesting to understand how p53 might regulate these phosphorylations. However, the conditional loss of FAK in v-Src-transformed p53+/+ MEF (Lingqiu Gao and I.H. Gelman, unpublished data; Hilary Beggs, UCSF, personal communication) or the ectopic expression of WT-p53 in our FAK-/-[v-Src] cells (Sanjay Sachdev and I.H. Gelman, unpublished data) leads to rapid apoptosis, making this determination technically unfeasible at the present time. In sum, our data strongly suggest that FAK- and adhesion-signaling can modulate which substrates are favored (or disfavored) by Src, thereby influencing the various dynamic biologies that contribute to cancer progression.

In regards to mechanism, we speculated previously that regulation of cytoskeletal dynamics by FAK and/or adhesion-activated signals might affect co-localization of Src with its potential substrates. Alternatively, even if FAK/Src complexes are found at the subcellular sites of potential substrates, such as focal adhesion plaques, the preferential binding of FAK to the Src-SH2 domain through its poY397 site may shift substrates to other, weaker binding sites on Src, such as the SH3 domain, thereby resulting in lower levels of phosphorylation [30].

There are very few studies that identify an obligate role for FAK in enhancing Src-mediated phosphorylation of specific substrates. For example, Wu et al. [29] demonstrated that full phosphorylation of endophilin A2 by Src requires FAK. Brown et al. [46] show that Src and FAK cooperate to phosphorylate PKL during cytoskeletal remodeling. El Annabi et al. [47] describe a system in which insulin-mediated receptor activation and IRS-1 tyrosine phosphorylation is enhanced in cells adhering to fibronectin versus suspended cultures, and that phosphorylation can be restored in suspended cells by the overexpression of both FAK and Src. Ruest et al. [48] showed that maximal phosphorylation of p130CAS in COS-7 cells required co-expression of Src and FAK. Roy et al. [28] showed that the ability of v-Src to phosphorylate the FAK family member, Pyk2, is attenuated by FAK, whereas the Src-induced phosphorylation of CAS and paxillin is enhanced by FAK. Hsia et al. [49] and Moissoglu et al. [30] identify another example where a potential Src substrate, STAT3, required neither FAK nor adhesion for maximal phosphorylation.

The current study is the first to describe either positive or negative roles for FAK and adhesion in the Src-induced phosphorylation of PKC-δ, p190RhoGAP, Shc, p120 catenin, connexin-43, cortactin, p56Dok-2, Crk, Eps8, PLC-γ1, Sam68 and vinculin. Our data indicate that FAK and/or adhesion status affect the phosphorylation of most of these substrates.

Phosphorylation of PKC-δ at Y311 by Src is associated with caspase-mediated apoptosis in many cell types (reviewed in ref. [50]). Our data showing increased PKC-δ^Y311 ^phosphorylation in adherent FAK-/-[v-Src] cells, whereas phosphorylation in suspension cells is FAK-independent, suggests that the ability of FAK to suppress apoptosis [1-4] is enhanced by integrin-mediated signals.

The Src-induced phosphorylation of Shc, PLC-γ1 and Eps8 was unaffected by FAK or adherence state. Phosphorylation of PLC-γ1 at Y783 is specifically induced by integrin-mediated adhesion, and mutation of this site antagonizes adhesion [51]. However, Tyorogov et al. [52] showed that adhesion-induced PLC-γ1^poY783 ^could occur in FAK-deficient MEF, in line with our findings that Src-induced PLC-γ1^poY783 ^is FAK-independent.

The Src-induced phosphorylation of p190RhoGAP and Crk is enhanced slightly in the absence of FAK irrespective of adherence state. Although p190RhoGAP can bind to and be phosphorylated by FAK [53,54], our data indicate that the v-Src-induced phosphorylation of p190RhoGAP is not dependent on FAK or adhesion. p190RhoGAP phosphorylation by v-Src is known to activate its ability to suppress RhoA, thereby inhibiting the formation of actin stress fibers and focal adhesion complexes in transformed cells [55]. Moreover, v-Src facilitates increased complex formation between p190RhoGAP and p120RasGAP.

In contrast, FAK may play a critical role in regulating p190RhoGAP activity in *untransformed *cells. Specifically, Y31/118-phosphorylated paxillin has been shown to liberate p190RhoGAP from complexes with p120 RasGAP, thereby allowing p190RhoGAP to suppress RhoA-mediated cytoskeletal remodeling in endothelial cells [56]. Indeed, our current data show that the relative levels of paxillin^poY31/118 ^are decreased in FAK-/- versus FAK+/+ MEF (i.e.- not expressing v-Src, Fig. 3), suggesting that FAK is required for p190RhoGAP-mediated inhibition of RhoA.

The Src-induced phosphorylation of p130CAS and vinculin was enhanced by FAK but not by adherence state. For example, phosphorylation of the Crk-associated substrate, p130CAS, by v-Src was favored slightly in FAK-expressing cells even though its ability to be phosphorylated at lower levels by v-Src in adherent FAK-/- cells [27,28] may be the result of adhesion-activated Pyk2 [57]. Our current results, that Src-induced phosphorylation of CAS at Y410 in adherent culture is enhanced by FAK, are similar to those of Roy et al. [28], though they looked at the overall tyrosine phosphorylation of CAS. In regards to biologic significance, the anchorage-independent tyrosine phosphorylation of CAS correlates with suppression of anoikis [58]. Src-mediated tyrosine phosphorylation of CAS is required for the invasive phenotype and metastasis formation but not for primary tumor growth [59]. Our present observation of enhanced phosphorylation of CAS in the presence of FAK, irrespective of adhesion, argues that FAK may positively influence the ability of Src to suppress anoikis and induce invasion and metastasis. Moreover, Patwardhan et al. [60] observed that CAS, independent of its phosphorylation status, enhances the ability of Src to promote anchorage-independent growth. Thus, taken with our data, we can conclude that differential CAS phosphorylation cannot be responsible for enhanced AIG we detect in the absence of FAK.

The FAK-dependent enhancement of Src-induced vinculin phosphorylation (at both Y100 and Y1065) irrespective of adherence state conflicts somewhat with the previous findings of Chang et al. [61] who showed that anoikis induced by FAK^Y397F ^expression in v-Src-transformed MEF only correlated with decreased CAS, but not vinculin, talin or paxillin protein levels. It should be stressed, though that the FAK^Y397F ^allele is not equivalent to a FAK-null condition in that i) FAK can bind v-Src (but not c-Src) through SH3-mediated interactions [62], and ii) FAK^Y397F ^may facilitate anoikis by acting as a sink for other survival factors.

The ability of FAK to either enhance or attenuate Src-induced phosphorylation of paxillin, p120catenin, connexin and p56Dok-2 was influenced by adherence state. We found that whereas Src-induced paxillin^Y118 ^phosphorylation is attenuated by FAK, FAK is required for optimal phosphorylation of paxillin^Y31 ^in suspension cultures. Interestingly, Roy et al. [28] and our lab [30] showed previously that the overall level of Src-induced paxillin tyrosine phosphorylation level is not affected by FAK, yet we showed that FAK-/-[v-Src] cells lacked some of the super-phosphorylated, slow-mobility paxillin isoforms found in FAK+/+[v-Src] cells. Our current work makes use of the site-specific phospho-Abs to show that FAK can either induce or attenuate paxillin phosphorylation at specific residues.

Our data show that FAK enhances Src-induced p120catenin^poY228 ^in adherent cultures yet attenuates Src-induced p120catenin^poY228 ^in suspension cultures. The tyrosine phosphorylation of p120catenin is associated with the loss of catenin-cadherin cell-cell adherens junctions in several systems [63,64]. The preponderance of data identifies functions for FAK in focal adhesion complexes, and not in cell-cell junctions. Not surprisingly, only one study indirectly links FAK with cell-cell junctions: Irby and Yeatman [65] show that FRNK, a naturally-occurring dominant-interfering FAK allele, can restore some of the Src-suppressed cell-cell interactions. This suggests that FAK functions downstream of Src to facilitate catenin phosphorylation, a conclusion backed by our findings. Nonetheless, p120catenin^poY228 ^levels do not correlate with changes in cell-cell interactions under suspension conditions with our MEF. How FAK might associate with or antagonize the phosphorylation of catenins in the absence of adherence signaling is unclear at this time. One hint may come from the finding that p120catenin translocates from cell-cell junctions to the cytoplasm during epithelial to mesenchymal transition and that cytoplasmic p120catenin correlates with poor prognosis and lymph node metastasis in colon cancer [66].

Phosphorylation of p56Dok-2 at Y351 establishes a binding site for the SH2 domain of adaptor protein Nck, which is involved in the organization of actin cytoskeleton [67-69]. Noguchi et al. [70] observed no appreciable difference between adherent versus suspension cultures of v-Src transformed rat fibroblasts in regards to Dok-1 phosphorylation level. However, the current study is the first to show that p56Dok-2 phosphorylation by Src is enhanced by FAK in adherent cells but attenuated by FAK in suspension cells. The significance of our observation remains unclear at this point but suggests that FAK can positively or negatively influence actin cytoskeletal dynamics through p56DOK-2 phosphorylation depending on adherence state.

We previously demonstrated that the AIG induced by Src in MEF was enhanced 5- to 10-fold in the absence of FAK [27]. The phosphorylation of two Src substrates, paxillin (at Y118), and p120catenin (at Y228), was increased in suspended FAK-/-[v-Src] cells relative to cells expressing FAK and growing in adherent conditions. Our data strongly suggest that paxillin^poY118^, but not p120catenin^poY228^, regulates the enhanced AIG phenomenon. This is the first identification of a required role for a specific paxillin phosphorylation event in AIG, and adds to recent evidence correlating paxillin phosphorylation at Y118 with AIG in colon cancer cells forced to express β_4_GalNAc-T3 [71] and the requirement for paxillin for epidermal growth factor-induced AIG growth of JB6 Cl41 fibroblasts [72]. A central role for paxillin^poY118 ^in driving AIG is underlined by our finding that two human colon cancer epithelial cell lines, HT25 and RKO, exhibit increased AIG after siRNA-mediated FAK knockdown, correlating with increased relative paxillin^poY118 ^levels under suspension growth conditions.

We showed previously that PI3K/AKT is a major mediator of Src-induced enhanced AIG in the absence of FAK [30]. Thus, FAK seems to attenuate the ability of Src to bind to and/or phosphorylate paxillin at Y118 (this study) and the p85 regulatory subunit of PI3K [30], resulting in decreased AIG. It is unclear whether paxillin and PI3K control common AIG-inducing pathways; the possibility that there is crosstalk between these two mediators has not been described in the literature to date.

## Conclusion

Our data identify FAK- and/or adhesion-regulated effects on the choice of substrates phosphorylated by Src. As oncogenic transformation and progression are multi-step processes, it will be interesting to determine how the FAK and adhesion effects we identified might correlate with the dynamic changes to the dependence on motility, cell survival and adhesion required at different points of cancer progression.

## Abbreviations

Ab: antibody; BS: bovine serum; DMEM: Dulbecco's modified Eagle's medium; FAK: focal adhesion kinase; GFP: green fluorescent protein; GST: glutathione S-transferase; IB: immunoblotting; IP: immunoprecipitation; PI3K: phosphatidylinositol 3-kinase; PVDF: polyvinylidene fluoride.

## Competing interests

The authors declare that they have no competing interests.

## Authors' contributions

SS and YB contributed equally to performing the experiments and preparing the manuscript. IHG, the Senior/Corresponding Author, conceived and oversaw the execution of the included experiments, and wrote and edited the manuscript.

## Pre-publication history

The pre-publication history for this paper can be accessed here:

http://www.biomedcentral.com/1471-2407/9/12/prepub
